# Prenatal DEHP exposure predicts neurological disorders via transgenerational epigenetics

**DOI:** 10.1038/s41598-023-34661-3

**Published:** 2023-05-06

**Authors:** Mita T. M. T. Tran, Fu-Chen Kuo, Jie-Ting Low, Yu-Ming Chuang, Sofia Sultana, Wen-Long Huang, Zhe-Young Lin, Guan-Ling Lin, Chia-Fang Wu, Sih-Syuan Li, Jau-Ling Suen, Chih-Hsing Hung, Ming-Tsang Wu, Michael W. Y. Chan

**Affiliations:** 1 Department of Biomedical Sciences, National Chung Cheng University, 168 University Road, Min-Hsiung, Chia-Yi, 621 Taiwan; 2 Epigenomics and Human Disease Research Center, National Chung Cheng University, Min-Hsiung, Chia-Yi, Taiwan; 3 Center for Innovative Research on Aging Society (CIRAS), National Chung Cheng University, Min-Hsiung, Chia-Yi, Taiwan; 4 Department of Chemical Engineering, National Chung Cheng University, Min-Hsiung, Chia-Yi, Taiwan; 5 Research Center for Precision Environmental Medicine, Kaohsiung Medical University, Kaohsiung, Taiwan; 6 School of Medicine, College of Medicine, I-Shou University, Kaohsiung, Taiwan; 7 Department of Obstetrics & Gynecology, E-Da Hospital, Kaohsiung, Taiwan; 8 International Master Program of Translational Medicine, National United University, Miaoli, Taiwan; 9 PhD Program in Environmental and Occupational Medicine, Kaohsiung Medical University, Room 721, CS Building, No.100, Shih-Chuan 1St Road, Kaohsiung, 807 Taiwan; 10 Department of Public Health, College of Health Sciences, Kaohsiung Medical University, Kaohsiung, Taiwan; 11 Department of Family Medicine, Kaohsiung Medical University Hospital, Kaohsiung Medical University, Kaohsiung, Taiwan; 12 Graduate Institute of Medicine, College of Medicine, Kaohsiung Medical University, Kaohsiung, Taiwan; 13 Department of Medical Research, Kaohsiung Medical University Hospital, Kaohsiung, Taiwan; 14 Department of Pediatrics, Kaohsiung Municipal Hsiao-Kang Hospital, Kaohsiung, Taiwan

**Keywords:** DNA methylation, Molecular medicine

## Abstract

Recent experimental and observational research has suggested that childhood allergic asthma and other conditions may be the result of prenatal exposure to environmental contaminants, such as di-(2-ethylhexyl) phthalate (DEHP). In a previous epidemiological study, we found that ancestral exposure (F0 generation) to endocrine disruptors or the common plasticizer DEHP promoted allergic airway inflammation via transgenerational transmission in mice from generation F1 to F4. In the current study, we employed a MethylationEPIC Beadchip microarray to examine global DNA methylation in the human placenta as a function of maternal exposure to DEHP during pregnancy. Interestingly, global DNA hypomethylation was observed in placental DNA following exposure to DEHP at high concentrations. Bioinformatic analysis confirmed that DNA methylation affected genes related to neurological disorders, such as autism and dementia. These results suggest that maternal exposure to DEHP may predispose offspring to neurological diseases. Given the small sample size in this study, the potential role of DNA methylation as a biomarker to assess the risk of these diseases deserves further investigation.

## Introduction

The human placenta plays a crucial role in regulating developmental programs essential to proper fetal growth^1^. Environmental factors, such as endocrine disruptors, can shape their functions throughout the prenatal stage and promote the development of inflammatory diseases (e.g. asthma) and developmental-related disorders, through epigenetic modifications^2–4^.

Plasticizers are commonly used in the synthesis of polyvinyl chloride to make packaging materials, such as those used for cosmetics, shampoo, and food. Some plasticizers, such as di-(2-ethylhexyl) phthalate (DEHP), are also endocrine disruptors^5^. Since DEHP is non-covalently bound to plastics, its probability of leaching is very high, thereby allowing entry into the human body via inhalation, ingestion, or dermal contact.

In a previous study, we reported that environmental endocrine disruptors (e.g., phthalate) increase fetal susceptibility to allergic diseases, via the neonatal immune system, including dendritic cells (DCs) and T cells. Mechanistic analysis revealed that paternal DEHP exposure can lead to the hypomethylation of the *igf2r* promoter with corresponding transgenerational epigenetic effects in mice from generations F1 to F4^4^.

Epigenetic modifications, including DNA methylation, histone modification, and microRNA expression, play important roles in normal cellular differentiation and the development of various human diseases. It is widely acknowledged that aberrant epigenetic modifications play a role in the development of cancer in humans. It has also been determined that these modifications can be induced by environmental factors. One previous study reported that exposure to phthalate can induce childhood asthma by altering DNA methylation^6^. Recent research has also indicated that changes in DNA methylation can be induced trans-generationally^4,6^.

In this study, we analyzed the dose-dependent effects of DEHP on DNA methylation in placental tissue. Our results revealed DNA hypomethylation in the high DEHP exposure group. This study also sought to elucidate the mechanisms by which prenatal phthalate exposure predisposes a child to neurological diseases through the modification of DNA methylation.

## Results

### Genome-wide DEHP-induced DNA hypomethylation in human placenta samples

**Figure 1:**
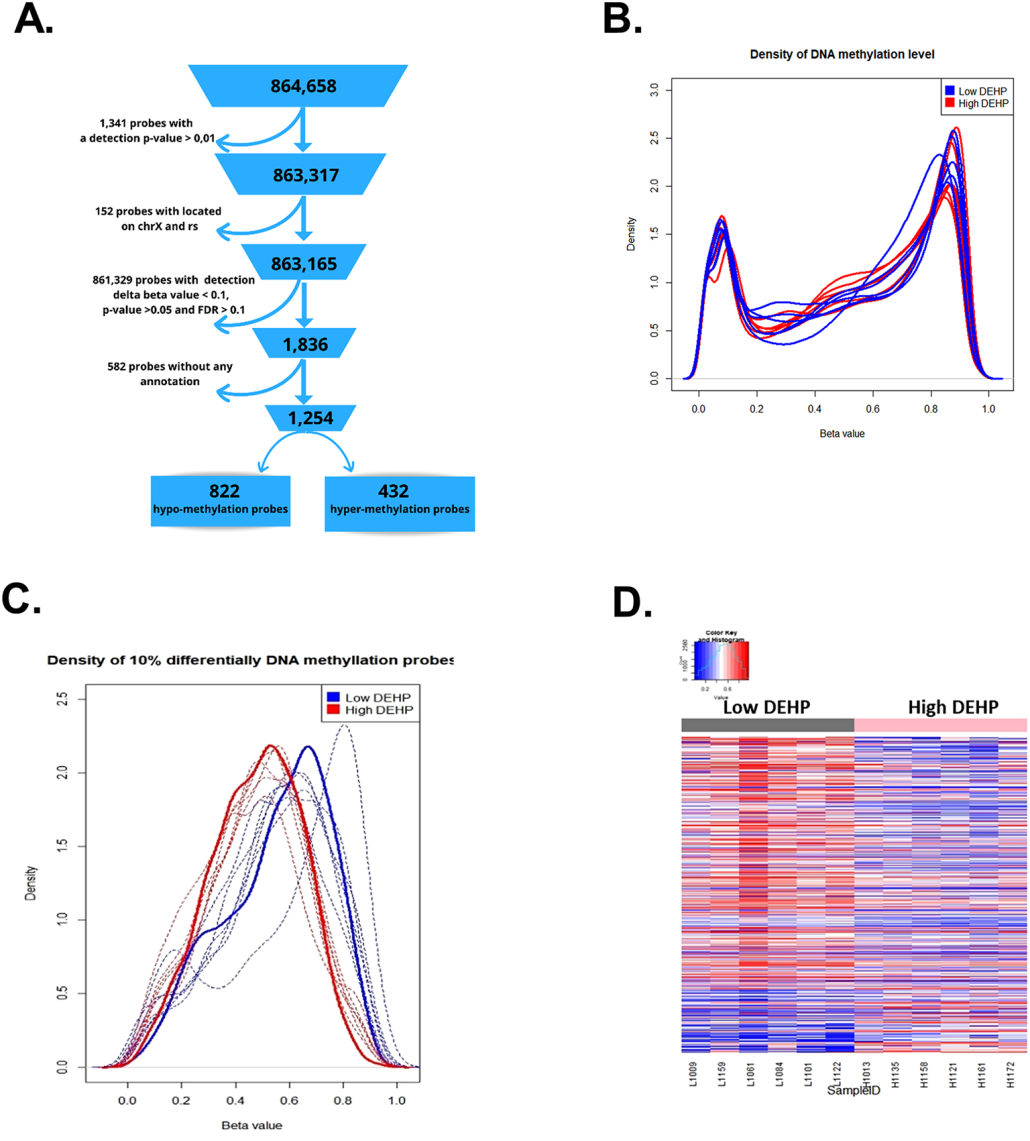
Infinium methylation microarray analysis of Methylation changes in DEHP-exposed human placenta samples. (**A**) The flowchart shows how the pipeline of raw data was processed. DNA methylation was performed using the high-resolution Infinium MethylationEPIC BeadChip Kit interrogating about 850,000 methylation sites quantitatively across the genome at single-nucleotide resolution. By utilizing a cut-off p-value threshold of greater than 0.05 and an FDR of 10%, a total of 1254 probes displaying a minimum of 10% differential methylation were identified. (**B**) Density of DNA methylation level for 12 human placenta samples exposed to DEHP; blue lines: low DEHP exposure (n = 6), red lines: high DEHP exposure (n = 6). Individual probes with beta (β)-values (range 0–1) are approximate representations of the absolute methylation percentage of specific CpG sites within the sample population. Beta value = 1 indicates complete methylation; beta value = 0 represents no methylation. (**C**) Density of 10% differentially DNA methylated probes of all samples. Hypomethylation was observed in the DEHP high group (red line), as compared to DEHP low group (blue line). (**D**) Heatmap showing the 10% differentially DNA methylated probes; left panel: low DEHP exposure; right panel: high DEHP exposure. Heatmap showing differentially methylated cytosine (DMC) sites across the two different exposure dosages of DEHP. Most of the probes observed were hypomethylation in DEHP (red line: methylated, blue line: unmethylated).

Infinium Human Methylation 850 K Beadchip was used to investigate the genome-wide DNA methylation of human placenta among 12 pregnant women exposed to DEHP (Table 1) (Fig. 1A). The distribution of beta values across all samples revealed a typical bi-modal distribution with two peaks close to 0 and 1 (Fig. 1B). Interestingly, among probes presenting at least 10% differences in methylation, hypomethylation was more pronounced in the DEHP-high group (red line) than in the DEHP-low group (blue line, Fig. 1C). This DEHP-induced hypomethylation appeared in the heatmap of differentially methylated cytosine (DMC, Fig. 1D). Taken together, we conclude that DEHP can induce DNA hypomethylation in human placenta tissue.

**Table 1:** Background data of 12 study subjects, including age, urinary concentrations of five DEHP metabolites, and estimated daily intake of DEHP during the third trimester.

ID	Age (years)	Five DEHP metabolites concentration in urine (ng/mL)					Urinary creatinine (mg/dL)	Ʃ DEHP metabolites conc. (μg/g Cre.)	DEHP exposure group
		MEHP	MEHHP	MEOHP	MECPP	MCMHP			
1–1	26	31.94	151.12	123.19	148.64	17.38	50.1	472.27	High
1–2	32	160.99	445.80	324.07	425.28	58.63	75.6	1414.77	High
1–3	32	102.89	480.64	371.50	390.16	33.79	91.6	1378.98	High
1–4	37	281.18	440.75	299.40	540.64	30.26	110.3	1592.23	High
1–5	37	470.45	25.25	28.37	32.79	5.21	23.6	562.07	High
1–6	40	5.21	61.38	67.55	405.91	189.09	36.1	729.14	High
Mean ± SD (GM, IQR)	34.00 ± 5.02 (33.67, 7.25)	175.44 ± 175.07 (84.52, 303.24)	267.49 ± 210.69 (167.54, 402.16)	202.35 ± 146.66 (142.88, 278.17)	323.90 ± 191.75 (237.06, 334.44)	55.73 ± 67.74 (31.76, 76.91)	64.55 ± 33.59 (33.67, 7.25)	1694.08 ± 504.63^1^ (1625.42, 791.92)	
2–1	26	1.92	7.42	8.40	11.48	3.77	86.8	32.99	Low
2–2	34	0.50	13.63	10.19	0.10	11.56	23.9	35.98	Low
2–3	34	1.82	9.78	8.35	14.34	4.60	89.2	38.89	Low
2–4	37	0.20	4.26	4.24	12.47	1.93	58.7	23.1	Low
2–5	37	0.18	13.36	11.19	12.72	2.74	65.6	40.19	Low
2–6	41	0.13	6.48	5.61	9.23	3.05	91.6	24.5	Low
Mean ± SD (GM, IQR)	34.83 ± 5.04 (34.50, 6.00)	0.79 ± 0.85 (0.45, 1.68)	9.16 ± 3.80 (8.45, 7.50)	8.00 ± 2.65 (7.58, 5.17)	10.06 ± 5.16 (5.37, 6.18)	4.61 ± 3.52 (3.85, 3.80)	69.30 ± 26.03 (63.45, 39.80)	59.92 ± 45.79^1^ (50.24, 48.39)	

*Cre.* creatinine, *DEHP* di-(2-ethylhexyl) phthalate, *GM* geometric mean, *IQR* inter-quartile range, *MCMHP* mono(2-carboxymethylhexyl)phthalate, *MECPP* mono(2-ethyl-5-carboxypentyl)phthalate, *MEHP* mono-(2-ethylhexyl) phthalate, *MEOHP* mono-(2-ethyl-5-hydroxylhexyl) phthalate, *MEHHP* mono-(2-ethyl-5-oxohexyl) phthalate, *SD* standard deviation.

^1^P-value = 0.004 for Mann–Whitney U Test and < 0.001 for log-transformation Student's t-statistic test.

### DEHP-induced methylation changes in the intergenic region

**Figure 2:**
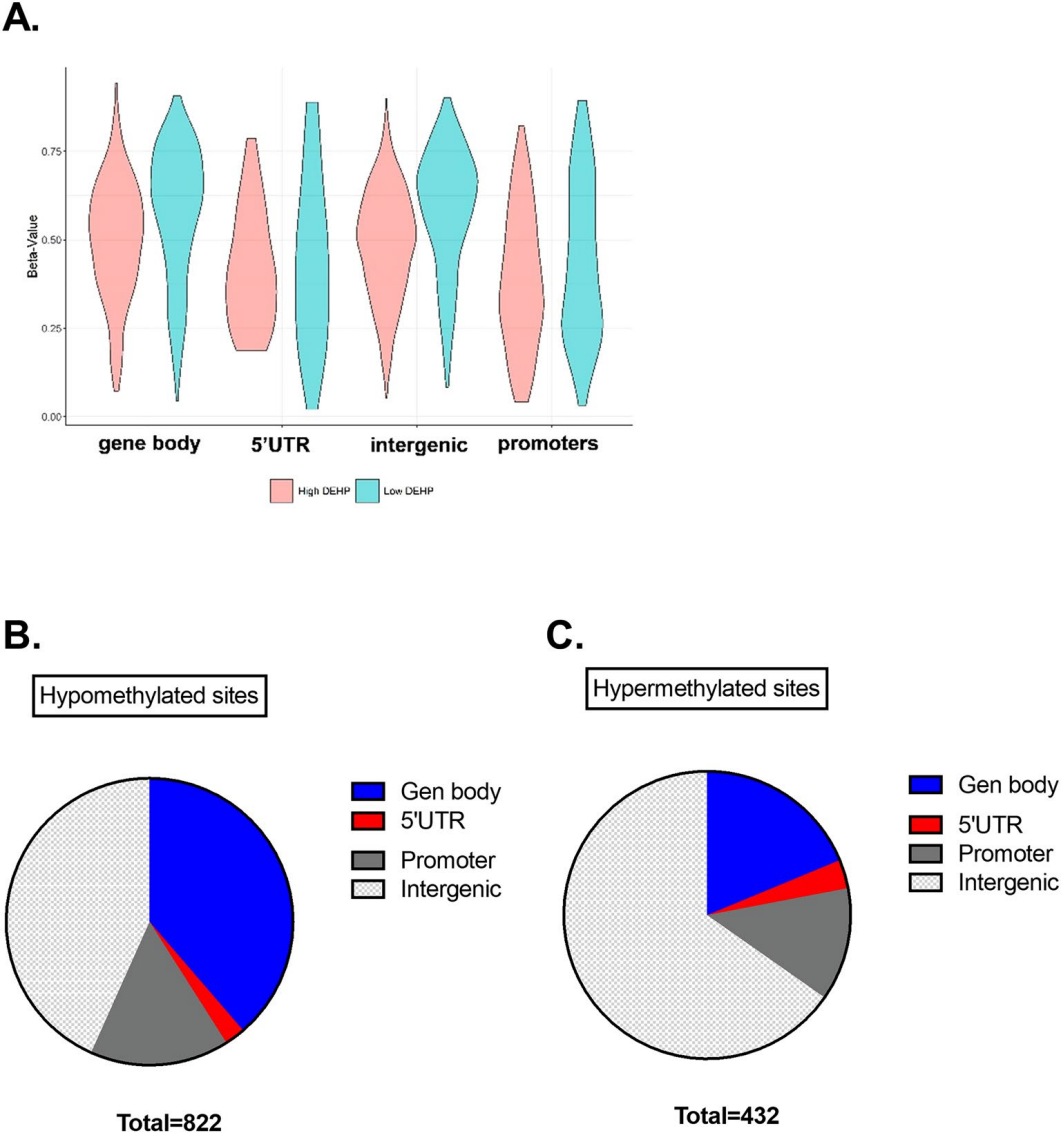
Spatial distribution of differentially methylated CpGs (DMCs) and gene-centric annotations. (**A**) Violin plot of 1261 probes showing beta values of gene body (n = 386), 5’UTR (n = 30), intergenic region (n = 677), and promoters (n = 168). The violin shape shows the quantitative distribution of DNA methylation. Pink denotes the high DEHP group, while blue denotes the low DEHP group. The distribution of (**B**) hypomethylated sites and (**C**) hypermethylated sites in the gene body, 5’UTR, intergenic region, and promoters is shown in the pie chart.

**Figure 3:**
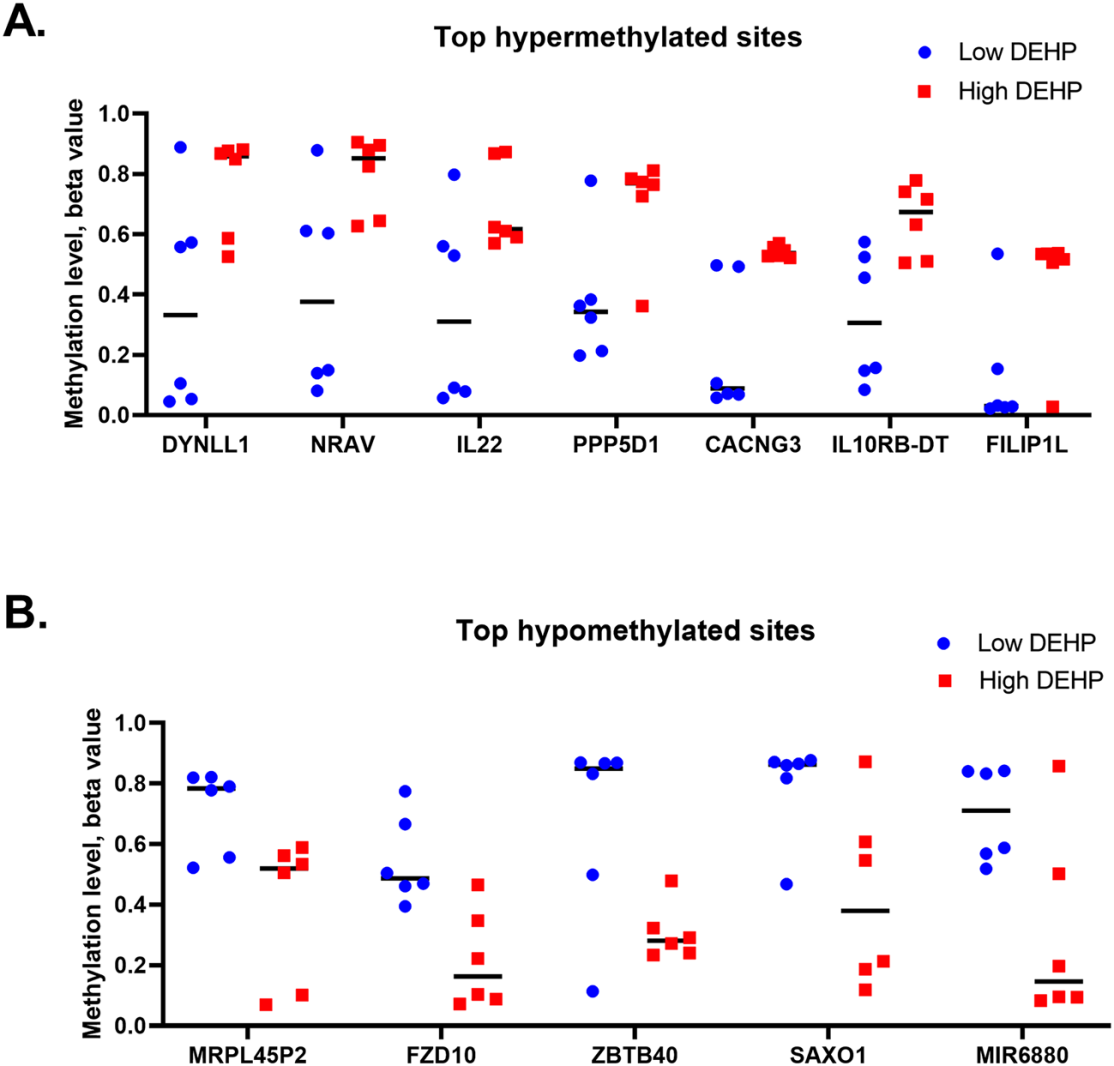
Top 30% differential methylated CpG sites between low and high DEHP-exposed groups. (A) Seven hypermethylated sites and (**B**) five hypomethylated sites with 30% DMC in high (blue) and low (black) DEHP-exposed groups are shown.

Further analysis was performed on genomic regions showing differential methylation under the influence of DEHP. Global hypomethylation was found in the gene body and intergenic region (Fig. 2A), in which most of the hypomethylated sites occurred (Fig. 2B). Note that sites of DNA hypermethylation were also observed in the gene body, intergenic region, and promoter region (Fig. 2C). The probes with the most pronounced hypomethylation were observed at 7 genes (Fig. 3A, Table 2), while those with the most pronounced hypermethylation were observed at 5 genes (Fig. 3B, Table 3).

**Table 2:** Top hypermethylation sites in human placenta exposed to DEHP.

TargetID	Δ Beta-value	Chromosome number	Position	P-value	Gene name	Distance from TSS	Annotation	Overlapping SNP
cg22138998	0.3942	12	120,903,935	0.0415	DYNLL1	3709	Gene body	No
cg26076233	0.3857	12	120,930,045	0.0347	NRAV	1905	Gene body	No
cg07493237	0.3369	12	68,619,796	0.0497	IL22	22,225	Promoters	No
cg02472644	0.3270	19	47,024,023	0.0150	PPP5D1	2088	Gene body	No
cg11074323	0.3265	16	24,298,532	0.0140	CACNG3	30,877	Gene body	rs2341934 (C > T)
cg11628320	0.3231	21	34,620,207	0.0133	IL10RB-DT	17,729	Promoters	rs2834161 (C > T)
cg20276377	0.3103	3	99,595,026	0.0247	FILIP1L	28,260	Gene body	no

**Table 3:** Top hypomethylated sites in human placenta exposed to DEHP.

TargetID	Δ Beta-value	Chromosome number	Position	P-value	Gene name	Distance from TSS	Annotation	Overlapping SNP
cg05828606	− 0.3210	17	45,481,057	0.0221	MRPL45P2	46,535	Gene body	No
cg01414116	− 0.3287	12	130,720,805	0.0040	FZD10	73,802	Intergenic	No
cg19154950	− 0.3686	1	22,740,395	0.0325	ZBTB40	37,948	Intergenic	rs7533770 (C > G/T)
cg13724111	− 0.3691	9	18,825,658	0.0293	SAXO1	101,989	Promoters	rs10963785 (C > A/T)
cg23878260	− 0.3934	12	124,827,315	0.0273	MIR6880	5589	Intergenic	no

We also analyzed the correlation between concentration of DEHP metabolites and methylation level of those differential methylated sites of the patients, showing a significant correlation (positive for hypermethylation and negative for hypomethylation) in most of the methylated sites, using linear (Fig. S1) or nonlinear regression (Fig. S2). These results further suggested that DEHP can induce a methylation changes in human placenta in a dose-dependent manner.

### Protein‑protein interaction (PPI) analysis

**Figure 4:**
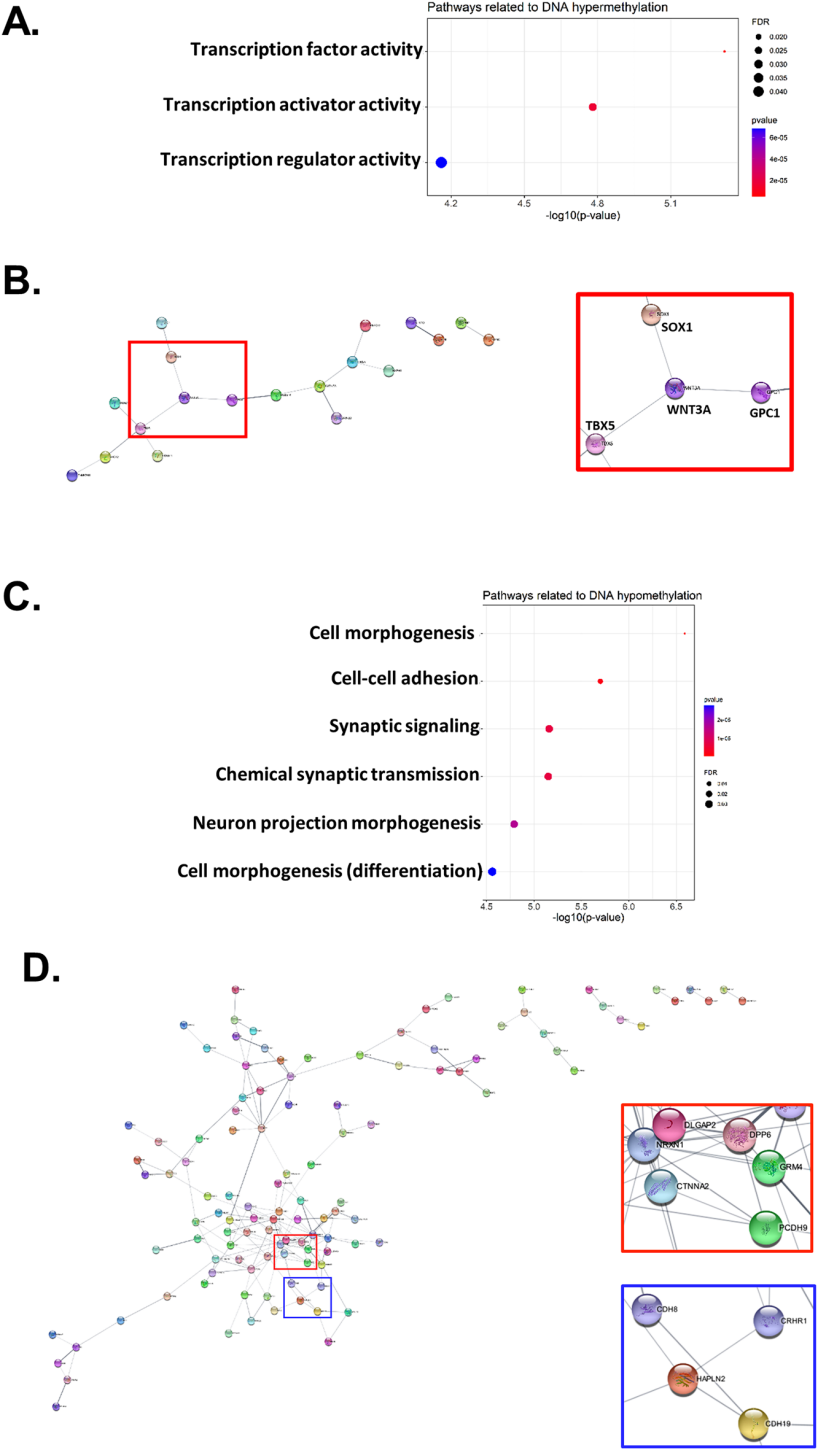
Cytoscape and STRING pathway analysis for genes with differential methylated CpG sites of human placenta exposed to low and high levels of DEHP. Pathways that are enriched with (**A**) hypermethylated genes and (**C**) hypomethylated genes are shown. Cytoscape analysis of protein–protein interaction (PPI) found that (**B**) hypermethylated genes are related to the Wnt signaling pathway, while (**D**) hypomethylated genes are related to neural-related adhesion molecules. The colored box was enlarged in the right panel. These figures on protein–protein interaction (**B,D**) were generated by Cytoscape version 3.9.1 (https://cytoscape.org/index.html)^36^.

A total of 598 genes, including 117 hypermethylated and 312 hypomethylated genes, were subjected to Cytoscape analysis of protein–protein interactions (PPIs, Fig. 4A–D, and Supplementary Table S1). Note that most of the proteins associated with DEHP-induced hypermethylation were involved in the transcriptional regulation of Wnt signaling (Fig. 4A,B), while most of the proteins associated with DNA hypomethylation were linked to adhesion molecules and neural networks (Fig. 4C,D).

### Changes in methylation in the imprinted region

**Figure 5:**
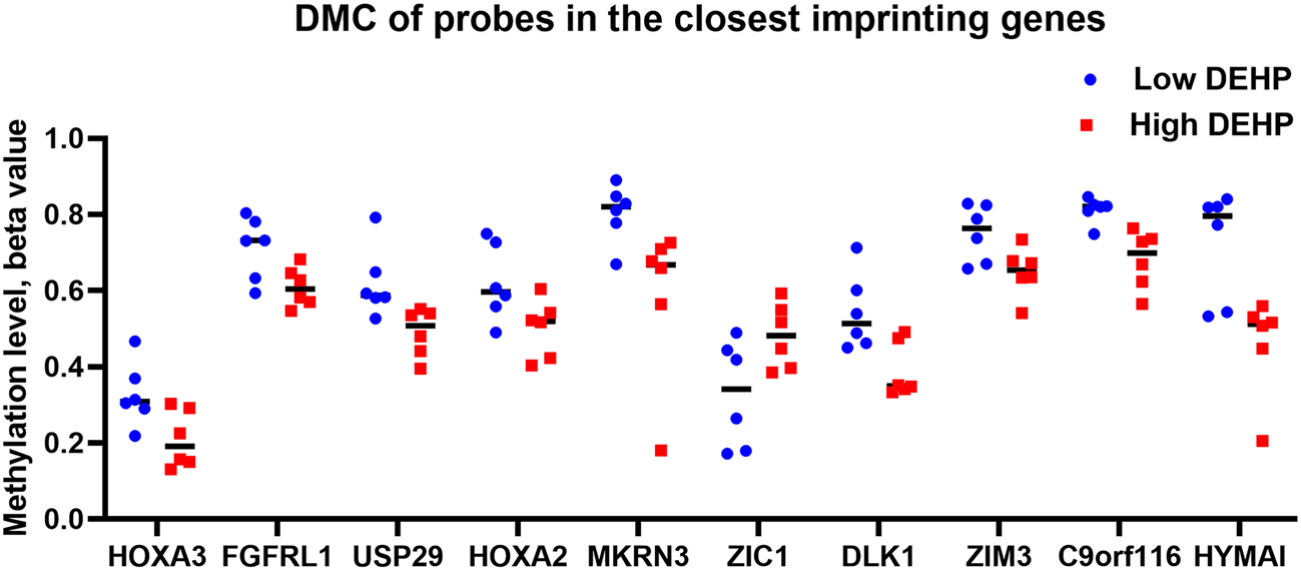
Histogram showing beta values of DMC probes located close to imprinting genes in high and low DEHP-exposed groups.

Shortly after fertilization (i.e. during preimplantation embryo development), the maternal genome undergoes passive demethylation, after which global remethylation corrects the embryonic methylation pattern. The differential methylation of 10 imprinted genes under exposure to DEHP was correlated with ECG abnormalities, autism, and alcohol dependence (Fig. 5, Table 4).

**Table 4:** Changes in DNA methylation in human placenta following exposure to DEHP.

TargetID	Δ Beta-value	Chromosome number	Position	P-value	Gene name	Distance from TSS	Annotation
cg00445443	− 0.1176	7	27,143,478	0.0286	HOXA3	2330	Intergenic
cg02163322	− 0.1034	4	1,017,759	0.0305	FGFRL1	11,508	Gene body
cg02399469	− 0.1298	19	57,631,118	0.0198	USP29	390	Promoters
cg04481096	− 0.1180	7	27,142,100	0.0459	HOXA2	2128	Gene body
cg06568768	− 0.2181	15	23,814,371	0.0492	MKRN3	3551	Gene body
cg16636671	0.1537	3	147,126,403	0.0497	ZIC1	748	Promoters
cg18092977	− 0.1524	14	101,190,167	0.0145	DLK1	3034	Intergenic
cg20414687	− 0.1022	19	57,646,348	0.0304	ZIM3	885	Gene body
cg21213388	− 0.1312	9	138,387,515	0.0065	C9orf116	489	Intergenic
cg23541304	− 0.2604	6	144,329,726	0.0083	HYMAI	3674	Promoters

## Discussion

The primary modulator of the intrauterine environment is the placenta, the function of which can be shaped by exposure to environmental contaminants, resulting in epigenetic alterations linked directly to fetal abnormalities^7–9^. Numerous studies have reported strong correlations between alterations in DNA methylation in the human placenta and abnormalities in the growth of the fetus^10–12^. One previous study reported that exposure to phthalate could have adverse effects on epigenetic outcomes in the human placenta^13^. Ravaei et al. recently reported that placental DNA methylation profiles can serve as a biomarker to predict the development of autism spectrum disorder (ASD) in fetuses^14^. Our current results confirmed that DEHP can indeed affect fetal development via aberrant DNA methylation.

Our previous study provided a convincing epigenetic explanation supporting a causal link between maternal exposure to DEHP and the transgenerational risk of allergic lung inflammation in offspring^4^. In the current study, we gauged the extent of the epigenetic effects of maternal DEHP exposure by examining global DNA methylation in 12 pregnant women exposed to DEHP. Our results were consistent with the findings in previous studies^4,15,16^, indicating that exposure to DEHP induced DNA hypomethylation in the placenta in a dose-dependent manner. Although the methylation levels of the CG sites seem to clusters at 0, 0.5 and 1 (Fig. 3), this phenomenon should not be caused by genetic effect, as only four probes (out of 12) were found to be overlapped with known SNP (Tables 2 and 3). On the contrary, correlation analysis using both linear and nonlinear regression demonstrated a significant correlation between concentration of DEHP metabolites and methylation level of the probes in most of the patients (Figs. S1 and S2), further indicating that DEHP affects DNA methylation in human placenta.

It is also important to note that most of the differential changes in methylation were observed in the gene body, promoter, and intergenic region. Note also that the genes associated with DEHP-induced hypermethylation were involved in transcriptional activity, particularly Wnt signaling, whereas the genes associated with DEHP-induced hypomethylation were linked to adhesion molecules and neural networks.

Wnt signaling plays an important role in cell proliferation and cell fate determination. Several developmental diseases in humans have been linked to alterations in Wnt signaling induced by environmental disruptors (e.g. phthalate)^17^. Zhang et al*.* reported that exposing pregnant rats to phthalate led to the down-regulation of Wnt/β-catenin signaling in fetal genital tubercles (GTs), indicating that phthalate may affect GT development^18^. Note that early exposure to phthalate has been linked to the development of neurological conditions, such as autism and dementia^19–22^. Those conditions have also been linked to the downregulation of Wnt signaling^23–25^; however, researchers have yet to elucidate the mechanism by which Wnt signaling is suppressed. Our findings in the current study suggest that Wnt signaling in the placenta is down-regulated by DEHP-induced hypermethylation. It also appears that these effects increase the susceptibility of the fetus to the development of these conditions.

Adhesion molecules associated with the cytoskeleton (e.g., cadherin) have also been shown to play an important role in maintaining the structural stability of neurons and preserving brain function^26^. Researchers have linked the dysregulation of adhesion molecules to the development of autism^27,28^ and dementia^29,30^. Maternal exposure to phthalate has been shown to upregulate cadherin levels in rodent models^31,32^. Our results in the current study suggest that the hypomethylation of adhesion molecules and cadherin following maternal exposure to DEHP may predispose the fetus to a wide spectrum of neurological disorders. These findings are consistent with a recent animal study in which juvenile exposure to phthalate was shown to exacerbate autism-like behavior via DNA hypomethylation^16^.

This study was subject to several limitations, which should be considered in the interpretation of the results. First, the sample size was small. Our findings will require confirmation using a large study cohort. Considerable effort was exercised in the selection of study subjects and age-matched controls to minimize the aging effect on DNA methylation. In addition, we excluded subjects with a history of cigarette smoking or environmental exposure to tobacco. Nonetheless, other lifestyle and environmental factors (e.g. diet and air pollutants) could not be controlled. These factors must be considered potential confounders biasing our results in either direction (i.e. under- or over-estimating the findings).

In conclusion, this study revealed a possible relationship between exposure to DEHP and global DNA methylation in the human placenta. This proof-of-concept study suggests that maternal exposure to DEHP predisposes the fetus to neurological disorders via epigenetic alteration. Future research with a larger sample size will be required to identify the mechanisms underlying the link between these effects and fetal abnormalities.

## Methods

### Human birth cohort and study design

This study was a part of the Taiwan Maternal and Infant Cohort Study (TMICS), a nationwide prospective birth cohort established by epidemiologists between October 2012 and May 2015. This study recruited pregnant women who visited one of nine hospitals (three in northern, three in central, two in southern, and one in eastern Taiwan) for routine pre-birth examinations during their third trimester (weeks 29 to 40). After providing written consent, the subjects were interviewed by nursing staff using a standardized questionnaire. Non-invasive biological specimens (blood, urine, and hair) were collected at the same time. Following the birth of the babies, samples of cord blood and placental tissue were aliquoted and stored at − 70 °C for future research. Details pertaining to the study design can be found in previous articles^33,34^.

In the current study, 146 potential subjects were recruited from E-Da Hospital. Note that phthalate metabolite data were available for all candidate subjects, based on one-spot urine samples collected during the third trimester. We excluded all individuals with a history of cigarette smoking and/or environmental exposure to tobacco during pregnancy. The sum of five DEHP metabolites (corrected via urinary creatinine) was ranked from highest to lowest and divided into four quartiles. The five DEHP metabolites included the following: MEHP (mono-(2-ethylhexyl) phthalate), MEOHP (mono-(2-ethyl-5-hydroxylhexyl) phthalate), MEHHP (mono-(2-ethyl-5-oxohexyl) phthalate), MECPP (mono(2-ethyl-5-carboxypentyl)phthalate), and MCMHP (mono(2-carboxymethylhexyl)phthalate). Note that all analysis was performed at an internationally certified laboratory (G-EQUAS 59) under the auspices of the National Health Research Institute (NHRI), as previously described^33^. We selected six subjects from the highest quartile and six subjects from the lowest quartile age-matched within two years during the third trimester of pregnancy. Table 1 lists the age distribution (26–41 years). Note that the mean sum of the 5 DEHP metabolites (μg/g creatinine) in the DEHP-high group (1694.08) was significantly higher than in the DEHP exposure group (59.92, P < 0.01, Table 1). This study was further approved by the IRB of E-Da Hospital, Taiwan (EMRP41101N (RI), EMRP31102N). Written informed consent was obtained from all study subjects, in accordance with the Declaration of Helsinki. All methods were performed in accordance with the relevant guidelines and regulations.

### DNA extraction

DNA was extracted using a Genomic DNA Mini Kit (Geneaid, Taiwan) in accordance with the manufacturer's instructions. DNA was then eluted in 50 µl of distilled water and stored at 0 °C until use.

### MethylationEPIC Beadchip analysis

Genome-wide methylation analysis was performed using the high-resolution Infinium MethylationEPIC BeadChip Kit, quantitatively interrogating roughly 850,000 methylation sites across the genome at single-nucleotide resolution. The GenomeStudio Methylation Module was used to facilitate the analysis of MethylationEPIC data. GenomeStudio Software was used to reveal valuable information, such as chromosomal coordinates, percentage of GC, location of the CpG island, and methylation $\beta$-values. Individual probe $\beta$-values (range 0–1) are approximate representations of the absolute methylation percentage of specific CpG sites within the sample population. Beta ($\beta$) = 1 indicates 100% methylation, whereas $\beta$=0 indicates 0% methylation. The values were derived by comparing the ratio of intensities between the methylated and unmethylated alleles, using the following formula^35^:

$$\beta -value= \frac{Max(Signal \,M,\,0)}{Max\left(Signal \,U, \,0\right)+Max\left(Signal \,M, \,0\right)+100}$$

where signal M and signal U respectively indicate the array intensity values for the methylated and non-methylated alleles. Samples were processed using the Bioconductor package designed explicitly for Illumina data. The DNA methylation data have been deposited in the Gene Expression Omnibus (GEO) database under accession number GSE153475. To facilitate comparisons among DEHP groups, we focused exclusively on probes with a mean change in methylation level of 10% or more (0.1 in β-value). P-values of < 0.05 or FDR values of < 0.1 were deemed indicative of significant changes.

### Statistical analysis

All statistical analysis was performed using GraphPad Prism version 8.0 for Windows (GraphPad Software, La Jolla, CA, USA) or R statistical software (version 4.2.1; R Foundation for Statistical Computing, Vienna, Austria). The ggplot2 package was used to perform Student’s T test and FDR for comparison of methylation differences. A P-value of < 0.05 was considered significant. Protein–protein interaction networks were integrated with DNA methylation differential data using Cytoscape version 3.9.1 in conjunction with the STRING App^36^.

### Ethics

This study received approval from the IRB of E-Da Hospital, Taiwan (EMRP41101N (RI), EMRP31102N). Written informed consent was obtained from all study subjects in accordance with the Declaration of Helsinki. All operations were performed in accordance with the relevant guidelines and regulations.

## Supplementary Information

Supplementary Figures.

Supplementary Table S1.

## Data Availability

DNA methylation microarray data have been deposited in the Gene Expression Omnibus (GEO) database, under accession number GSE153475.
