# Supplementary material for: Prenatal DEHP exposure predicts neurological disorders via transgenerational epigenetics
Source: Sci Rep. 2023 May 6;13:7399. doi: 10.1038/s41598-023-34661-3 (PMC10164151; doi:10.1038/s41598-023-34661-3)
Supplement: Supplementary file 1 — Supplementary Figures. [file 41598_2023_34661_MOESM1_ESM.pdf]

A

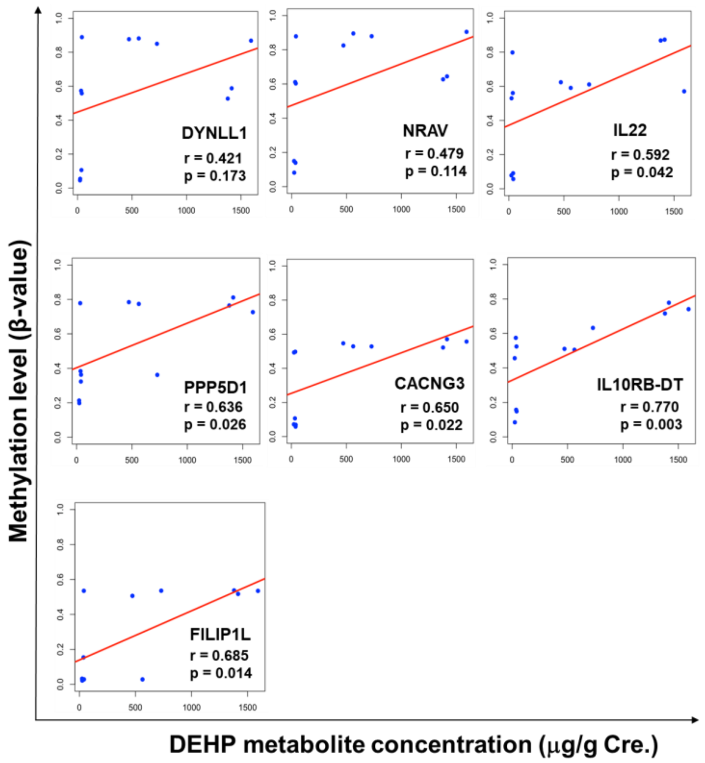

B

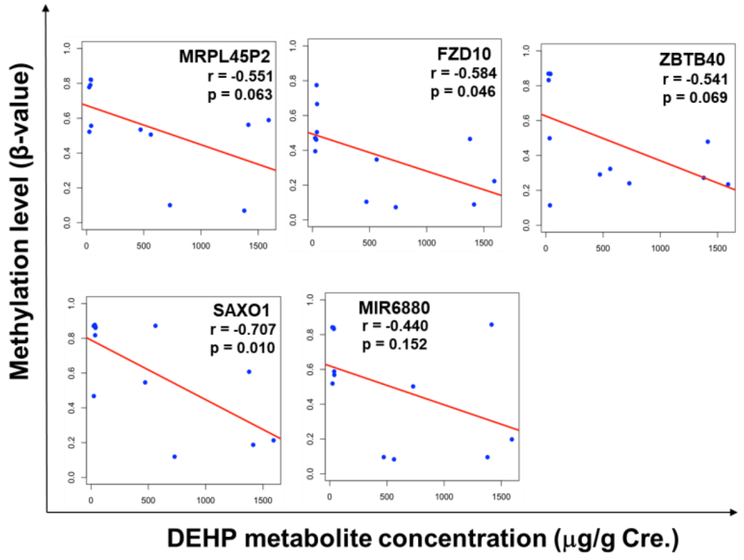

Supplementary Figure S1. Correlation between concentration of DEHP metabolites and methylation level of (A) top hypermethylation and (B) top hypomethylation sites of the patients. Linear regression was performed to analyze the correlation. Pearson correlation coefficient (r) and p value of each genes are shown.

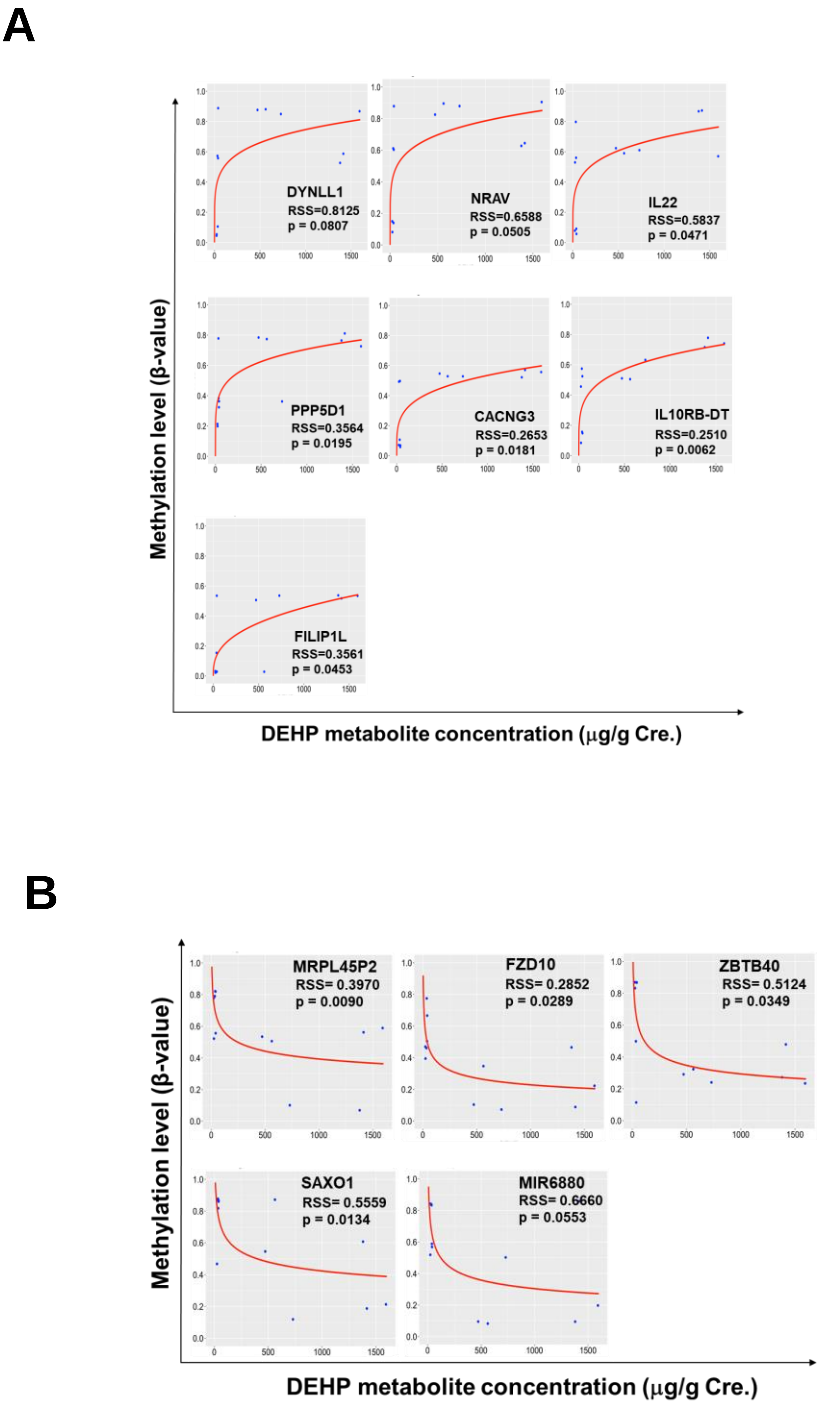

Supplementary Figure S2. Correlation between concentration of DEHP metabolites and methylation level of (A) top hypermethylation and (B) top hypomethylation sites of the patients. Nonlinear regression was performed to analyze the correlation. Residual Sum of Squares (RSS) and p value of each genes are shown.
